# Clinical value of hematologic test in predicting tumor response to neoadjuvant chemotherapy with esophageal squamous cell carcinoma

**DOI:** 10.1186/1477-7819-12-43

**Published:** 2014-02-25

**Authors:** Yinan Liu, Jinfeng Chen, Ningsheng Shao, Yuan Feng, Yuzhao Wang, Lijian Zhang

**Affiliations:** 1 Key laboratory of Carcinogenesis and Translational Research (Ministry of Education), Department of Thoracic Surgery II, Peking University Cancer Hospital & Institute, Beijing 100142, People’s Republic of China; 2 Department of Biochemistry and Molecular Biology, Beijing Institute of Basic Medical Sciences, Beijing 100850, People’s Republic of China

**Keywords:** Esophageal carcinoma, Neoadjuvant chemotherapy, Hematologic test

## Abstract

**Background:**

To investigate the relationship between hematologic test results and the predictive effect of regression of esophageal cancer after neoadjuvant chemotherapy (NACT), we analyzed pre-NACT hematologic data and their relationship to tumor regression.

**Methods:**

Thirty-eight consecutive patients with locally advanced squamous cell esophageal carcinoma who had undergone two cycles of paclitaxel/carboplatin NACT were enrolled. On the day prior to the first cycle of chemotherapy, hematologic tests, including routine blood test and biochemical examinations, were recorded. All patients were confirmed to have no history of hepatitis. Surgical resection was performed when clinical restaging showed effective regression. Histopathological examination was routinely performed to evaluate the postoperative effects of chemotherapy.

**Results:**

After two cycles of NACT, tumor imaging evaluation showed that 27 of the 38 patients had CR and PR, including 25 patients who underwent radical esophagectomies. Six patients had stable disease and five patients had progressive disease. According to the hematologic test results before NACT, patients with higher white blood cell counts, lymphocyte percentages, mononuclear cell counts, neutrophilic granulocyte counts, and eosinophilic granulocyte counts and lower alanine aminotransferase (ALT) level had a significantly greater opportunity for an effective response.

**Conclusion:**

Basal host immunologic function and hepatic function are associated with tumor response to NACT in patients with esophageal cancer. These parameters may have a certain predictive efficacy on NACT for esophageal squamous cell carcinoma.

## Background

Worldwide, almost 400,000 new cases of esophageal cancer are diagnosed annually. Esophageal cancer is the eighth most common cancer and the sixth most common cause of cancer-related mortality [1]. Esophageal squamous cell carcinoma (ESCC) is a major histological form of esophageal cancer in Asian countries. It is one of the most lethal malignancies of the digestive tract, and in most cases the initial diagnosis is established only once the malignancy reaches the advanced stage. For many years, the standard therapy for locally advanced lesions has been surgical resection. However, overall survival for patients with locally advanced tumors after resection remains poor, even in the minority with resectable disease for whom the 5-year survival rate ranges from 10% to 35% [2-4]. The last decade has seen the introduction of multimodal therapy regimens. Chemotherapy has become the standard first-line therapy for patients with advanced ESCC, particularly neoadjuvant chemotherapy (NACT) [5]. Histological tumor regression after chemotherapy is believed to be an important objective parameter and has been shown to have prognostic value in several studies. In clinical practice, patients who respond to preoperative treatment have significantly improved survival. However, the initial response rate for NACT remains at 35% to 66% [6] and non-responders are at risk for serious adverse effects, with no survival benefit.

It is therefore important to identify prognostic factors in esophageal cancer and determine which patients are most likely to respond to chemotherapy, preventing patients from undergoing ineffective chemotherapy and potentially toxic treatments. The published research [7-9] mainly focuses on the tumor itself, exploring oncologic special expression patterns or metabolism, such as biomarker levels and abnormal protein or gene expression, but ignores patient status in terms of its relationship with treatment efficacy. Although oncologic disease is a systemic disorder, research that focuses on the local tumor status, as above, cannot describe the integral anticancer situation. In particular, complicated laboratory methods cannot be easily transferred to economic methods of clinical evaluation in daily practice.

Considering the shortcomings of previous studies, we hypothesized that common routine hematologic tests might provide clinically useful information about the body status and reflect the tumor response to NACT. We retrospectively analyzed routine hematologic test results and investigated their relationship with the response to NACT.

## Patients and methods

### Eligibility criteria

From January 2012 to December 2012, a total of 38 patients with esophageal cancer who underwent NACT were enrolled in this study. This study was approved by the Medical Ethics Committee of Beijing Cancer Hospital. Written informed consent was obtained from the patients before the operations. All patients had biopsy-confirmed locally advanced, potentially curable ESCC. Mainly patients with tumors of clinical stage T1-2 N1 or T3N0-1 were enrolled. Patients with >10 cm extended mucosal lesions (T1-2) without local visible lymphatic metastasis (stage IA, IB) also underwent NACT in case of unresected microscopic metastases or possible R1-2 (microscopically or macroscopically positive margin) resection. Eligible patients were aged less than 75 years, had a World Health Organization (WHO) performance status score of 2 or lower, and had lost ≤10% of body weight. All patients also had adequate hematologic, renal, hepatic, and pulmonary function and no history of other cancer or previous radiotherapy or chemotherapy. Pretherapeutic staging procedures to confirm clinical-T3/4 (c-T3/4) categorization included endoscopy or endoscopic ultrasound and enhanced computed tomography (CT) of the chest and abdomen. None of the patients received concurrent radiation therapy before the operation.

### Analysis of hematologic test

Before the first cycle of neoadjuvant chemotherapy, hematologic tests, including routine blood test and biochemical examinations, were recorded to assess patients’ physical status. The biochemical examination includes hepatic function and renal function. The alanine aminotransferase (ALT) level is the most important factor in hepatic function. The exact time to perform hematologic testing was specified as between 1 day and 1 week before NACT. Moreover, we needed to exclude patients with, for example, fever or history of hepatitis.

### Treatment protocol

#### NACT

NACT comprised two cycles of paclitaxel (175 mg/m^2^) on day 1 followed by one cycle of carboplatin (AUC = 5) every 3 weeks. All patients were intravenously premedicated with dexamethasone, diphenhydramine, cimetidine, and standard antiemetic agents. The patients were closely monitored for toxic effects of chemotherapy with the use of the National Cancer Institute’s Common Terminology Criteria for Adverse Events, version 3.0.

#### Clinical restaging

Three weeks after the completion of two cycles of NACT, all patients routinely underwent clinical restaging to evaluate the reactive response with endoscopy and enhanced CT of the chest and abdomen according to the WHO evaluating criteria for chemotherapy. The chemotherapy response was defined as a complete response (CR), partial response (PR), stable disease (SD), or tumor progression (progressive disease (PD)).

#### Surgical resection

Patients with CR and PR underwent surgical resection 4 to 5 weeks following NACT. The acceptable approaches to resection included McKeown esophagectomy or Ivor Lewis subtotal esophagogastrectomy. Patients with SD and PD underwent radical chemoradiotherapy.

#### Histopathological examination

Histopathological examination of all resected specimens comprised thorough evaluations of tumor stage, residual tumor category (R category), grading, and lymph nodes status for an adequate assessment for the presence of residual tumor tissue and the effects of therapy.

### Statistical analysis

For group comparisons, unordered categorical variables were compared using Pearson’s chi-square (*χ*^2^) or Fisher’s exact test. The Student’s *t*-test or Mann–Whitney test was used to determine differences in continuous variables. Categorical variables are presented as percentage. *P* values < 0.05 were considered to be statistically significant. Statistical analysis was performed using SPSS software (version 18.0) for Windows (SPSS Inc., Chicago, IL, USA).

## Results

### Patient characteristics

The histological diagnosis was squamous cell carcinoma in all 38 cases. The patients had a mean age of 60 years (range, 37-70 years). There were five women and 33 men. Sixteen of the 38 patients (42.1%) had a smoking history of >20 years (Table 1). According to the TNM system, 35 lesions before NACT presented with a T2 or T3 extent of invasion. Three-quarters of the patients had tumors between 6 cm and 8 cm in diameter. Three patients with clinical stage T1 cancer demonstrated a diffuse pattern in mucosal lesions of >10 cm according to endoscopy or endoscopic ultrasound. All 38 patients underwent standard chemotherapy comprising two cycles of paclitaxel/carboplatin.

**Table 1:** Patient characteristics

		**Chemotherapy effect**		
		**CR + PR**	**SD + PD**	***P *** **value**
**Gender**				
	Male	22	11	0.161
	Female	5	0	
**Age (years)**				
	<60	12	5	0.617
	>60	15	6	
**Smoking history (years)**				
	<20	17	5	0.264
	>20	10	6	
**Differentiation**				
	Poor	8	4	0.546
	Moderate	14	5	
	Well	5	0	

### Regression response with respect to clinical evaluation

Investigation of the response based on the clinical evaluation results revealed no statistically significant differences in gender, age, or smoking history among all patients (*P* >0.05). Tumor differentiation grading (well, moderately, and poorly differentiated) based on the WHO classification system showed no association with tumor regression (*P* = 0.546) (Table 1).

After two cycles of NACT, tumor imaging restaging showed that 27 of the 38 patients had CR and PR, including 25 patients who received radical esophagectomies. Another two patients who showed PR had refused esophagectomy for personal reasons. Six patients had SD, one of whom strongly desired an operation; as we felt that this patient might benefit from surgery, we conducted an exploratory operation, and confirmed that the tumor had invaded the main aorta and that a radical resection was not possible. The other five SD patients received definitive chemoradiotherapy. Five patients had PD, who had lost the opportunity for operation and underwent definitive chemoradiotherapy (Table 2).

**Table 2:** Clinical restaging post NACT and histomorphological evaluation

		**Case (*n*)**	**%**
**Response to NACT (radiographic restaging)**			
	CR	3	7.9
	PR	24	63.2
	SD	6	15.8
	PD	5	13.2
**Histomorphological evaluation**			
	No vital residual tumor cells	2	5.3
	Decreased vital residual tumor cells with fibrosis	9	23.7
	No vital post chemotherapy change	14	36.8
	Metastasis post NACT	1	2.6

### Chemotherapy-related histomorphological findings

There were 26 patients underwent operations after NACT and 25 radical esophagectomy specimens, which were evaluated according to the standardized protocol. We evaluated the primary site with respect to morphological chemotherapy-induced changes. The tumor characteristic, including tumor location, tumor stage, and surgical mode were listed in Table 3. To determine the tumor regression and response to NACT, we evaluated the primary site with respect to morphological changes that have been previously described as typical of chemotherapy-induced changes in adenocarcinomas.^5^ Of the 38 patients, nine (23.7%) had obvious tumor regression, microscopically described as a decreased number of vital residual tumor cells, a centrifugal pattern with tumor-free fibrosis in the central and superficial luminal portions of the tumor bed, and areas of necrosis and inflammation. Two patients (5.3%) had pathological complete response (p-CR) and the microscopic description noted no evidence of vital residual tumor cells. Fourteen patients (36.8%) had minimal or no chemotherapy reaction in the pathological findings, although the tumor was diminished on imaging (Table 2).

**Table 3:** Characteristics of ESCC and surgical method

		**Case (*n*)**	**%**
**Location**			
	Proximal	10	26.3
	Middle	19	50
	Distal	9	23.7
**T stage**			
	T1	3	7.9
	T2	2	5.3
	T3	33	86.8
**c-TNM stage**			
	IA	2	5.3
	IB	1	2.6
	IIA	5	13.2
	IIB	6	15.8
	IIIA	9	23.7
	IIIB	12	31.6
	IIIC	3	7.9
**Surgical mode**			
	Ivor Lewis	17	44.7
	McKeown	8	21.1
	Patients refused	2	5.3
	Disease progression^a^	10	26.3
	Exploration^b^	1	2.6

^a^The 10 patients with c-restaging for PD and SD did not undergo operations.

^b^The patient with c-restaging for SD underwent exploratory thoracotomy without esophagectomy.

### Hematologic findings

A correlation between an effective response achieved at the end of treatment and different prognostic factors revealed that higher immunocyte counts may be associated with a better response to chemotherapy. Statistical analysis showed that patients with a higher white blood cell count (*P* = 0.003), lymphocyte percentage (*P* = 0.047), mononuclear cell count (*P* = 0.027), neutrophilic granulocyte count (*P* = 0.005), and eosinophilic granulocyte count (*P* = 0.038) had a better opportunity for an effective response (CR + PR) (Table 4). Meanwhile, there was a tendency for better results in patients with lower level of ALT. The CR + PR group patients had lower level of ALT compared with the SD + PD group (12.00 ± 3.99 *vs.* 18.00 ± 5.29, *P* = 0.003) (Table 4).

**Table 4:** Hematologic test before the first cycle of NACT for ESCC

	**CR + PR**	**SD + PD**	***P *** **value**
WBC (*10^9^/L)	6.80 ± 1.85	8.42 ± 1.10	0.003
Lym% (%)	26.28 ± 8.49	20.50 ± 6.86	0.047
MON# (*10^9^/L)	0.40 ± 0.19	0.60 ± 0.23	0.027
NEUT# (*10^9^/L)	4.54 ± 1.60	6.25 ± 1.40	0.005
EO# (*10^9^/L)	0.08 ± 0.06	0.13 ± 0.05	0.038
ALT (U/L)	12.00 ± 3.99	18.00 ± 5.29	0.003

## Discussion

NACT followed by surgical resection has become increasingly used in the treatment of locally advanced ESCC to improve patient outcomes [10-12]. This approach may increase local resectability rates and eliminate distant micrometastases because at least 50% of patients will experience a relapse of cancer arising from unresected microscopic metastases present at the time of surgery [13]. A meta-analysis of the survival benefit in the neoadjuvant setting revealed an increase in survival for patients with ESCC undergoing NACT [14]. In addition, the pathological response to preoperative chemotherapy has been shown to improve overall survival [15]. The regression percentage varies according to different investigators [7]. That of the primary tumor ranged from 59% to 87% in different preoperative randomized and non-randomized studies [7,16-18]. In our group, three of 38 (8%) patients achieved CR and 24 of 38 (63.1%) patients achieved PR, indicating that 27 of 38 (71.0%) patients were down-staged, similar to previous studies.

We obtained R0 resection in 97.2% (25/26) of patients who underwent resection. This R0 resection rate compares favorably with those reported in the literature, which are typically above 80% [19,20]. The current gold standard for response evaluation of esophageal tumors is histopathological assessment described by Mandard et al. [21]. There is a general consensus that patients who have achieved p-CR (ypT0N0M0) would benefit from NACT and would have better survival; most large randomized trials have demonstrated rates of p-CR ranging from 10% to 30% [22]. The p-CR rate of 5% (2 of 38 patients) in the present study is similar to the results of these trials.

There are several theoretical components available for the prediction of the pretherapeutic response [23-25]. In these influencing factors immunity state of patients is an important factor. Anti-tumor immunity consists of antigen-specific CD4+/CD8+ T cell immunity and humoral immunity. Chemotherapy can comprehensively influence anti-tumor immunity. Changes in lymphocyte percentage, neutrophil, eosinophil, and mononuclear cell counts arguably reflect the basic immune level. In the present cohort, we found that the hematologic cell level with respect to the basal host reaction, such as the white blood cell count, lymphocyte percentage, mononuclear cell count, neutrophilic granulocyte count, and eosinophilic granulocyte count, are related to the treatment efficacy. This may explain lymphocyte percentage, which could be considered a prognostic factor in NACT decision-making.

Existing pharmacokinetic and pharmacological distribution research has confirmed that paclitaxel is metabolized in the liver, via the biliary tract, and excreted mainly in the feces [2]. Early pharmacokinetic and disposition studies showed the importance of biliary excretion and hepatic metabolism in the clearance of chemotherapy drugs, which led to the concept of paclitaxel and carboplatin dose reductions in patients, based on hepatic dysfunction. In our cohort, ALT was deemed as an important indicator of hepatic function, and was negatively associated with the regression of NACT. This relationship indicated that better hepatic function was associated with metabolic capacity and pharmacological effect for the paclitaxel/carboplatin regimen, and the same compared dose will lead to better evaluation.

Effective markers for individualized chemotherapy regimens are an area of lively current research. So far, most researchers have focused on tumor cells and their molecular markers as predictors of chemotherapy outcomes or optimal regimens. However, these studies ignored the effects of patients’ general status on chemotherapy. Our research investigated the relationship between hematologic test and the therapeutic efficacy of NACT, and found that hematologic tests could, to a certain extent, predict appropriate chemotherapy. Our results show a complete new direction for individualized therapy of esophageal cancer. Combined with predictive markers, these hematologic tests may contribute powerfully to individualized therapy.

## Conclusion

Our retrospective analysis showed that hematologic test results with respect to basal host status and hepatic function were associated with response to NACT. Although our study was retrospective in nature, our conclusions have demonstrated that hematologic test results can reflect the body status and provide information regarding the potential response to NACT. This is a very important finding. We plan to design a prospective study to investigate the predictive efficacy of hematologic test on NACT for esophageal carcinoma.

## Consent

Written informed consent was obtained from the patients before operation.

## Abbreviations

ALT: Alanine aminotransferase; CR: Complete response; EO#: Eosinophilic granulocyte count; ESCC: Esophageal squamous cell carcinoma; LYM%: Lymphocyte percentage; MON#: Mononuclear cell count; NACT: Neoadjuvant chemotherapy; NEUT#: Neutrophilic granulocyte count; p-CR: Pathological complete response; PD: Progressive disease; PR: Partial response; R0: R1, R2, Residual tumor category (0, 1, 2); SD: Stable disease; WBC: White blood cell; WHO: World Health Organization.

## Competing interests

The authors declare that they have no competing interests.

## Authors’ contributions

YNL contributed to the preparation of the manuscript. NSS, YF, YZW, and LJZ participated in the design of the study and performed the statistical analysis. JFC conceived of the study, participated in its design and coordination, and helped to draft the manuscript. All authors read and approved the final manuscript.
